# Rad3^ATR^ Decorates Critical Chromosomal Domains with γH2A to Protect Genome Integrity during S-Phase in Fission Yeast

**DOI:** 10.1371/journal.pgen.1001032

**Published:** 2010-07-22

**Authors:** Sophie Rozenzhak, Eva Mejía-Ramírez, Jessica S. Williams, Lana Schaffer, Jennifer A. Hammond, Steven R. Head, Paul Russell

**Affiliations:** 1 Department of Molecular Biology, The Scripps Research Institute, La Jolla, California, United States of America; 2 Laboratory of Structural Biology, National Institute of Environmental Health Sciences, Research Triangle Park, North Carolina, United States of America; 3 DNA Array Core Facility, The Scripps Research Institute, La Jolla, California, United States of America; 4 Department of Cell Biology, The Scripps Research Institute, La Jolla, California, United States of America; The University of North Carolina at Chapel Hill, United States of America

## Abstract

*Schizosaccharomyces pombe* Rad3 checkpoint kinase and its human ortholog ATR are essential for maintaining genome integrity in cells treated with genotoxins that damage DNA or arrest replication forks. Rad3 and ATR also function during unperturbed growth, although the events triggering their activation and their critical functions are largely unknown. Here, we use ChIP-on-chip analysis to map genomic loci decorated by phosphorylated histone H2A (γH2A), a Rad3 substrate that establishes a chromatin-based recruitment platform for Crb2 and Brc1 DNA repair/checkpoint proteins. Unexpectedly, γH2A marks a diverse array of genomic features during S-phase, including natural replication fork barriers and a fork breakage site, retrotransposons, heterochromatin in the centromeres and telomeres, and ribosomal RNA (rDNA) repeats. γH2A formation at the centromeres and telomeres is associated with heterochromatin establishment by Clr4 histone methyltransferase. We show that γH2A domains recruit Brc1, a factor involved in repair of damaged replication forks. Brc1 C-terminal BRCT domain binding to γH2A is crucial in the absence of Rqh1^Sgs1^, a RecQ DNA helicase required for rDNA maintenance whose human homologs are mutated in patients with Werner, Bloom, and Rothmund–Thomson syndromes that are characterized by cancer-predisposition or accelerated aging. We conclude that Rad3 phosphorylates histone H2A to mobilize Brc1 to critical genomic domains during S-phase, and this pathway functions in parallel with Rqh1 DNA helicase in maintaining genome integrity.

## Author Summary

Eukaryotic genomes, which range in size from ∼10^7^ to ∼10^11^ base pairs, are replicated with nearly absolute fidelity every cell cycle. This amazing feat happens despite the frequent stalling or collapse of replication forks. The checkpoint kinase ATR is activated by replication fork stalling and phosphorylates histone H2A in nucleosomes surrounding damaged DNA. As the genomic regions triggering ATR activation are largely unknown, we used a whole-genome microarray to map chromosomal domains enriched with phospho-H2A during DNA replication in fission yeast. This analysis identified specific sites, including natural replication fork barriers in ribosomal DNA repeats, retrotransposon elements, and most surprisingly, all heterochromatin regions. Phospho-H2A binds the genome maintenance protein Brc1, and our genetic studies reveal that this molecular pathway becomes crucial in the absence of Rqh1, a conserved DNA helicase that is linked to cancer predisposition. As the fission yeast and human genomes share many similarities, our study reveals genomic landmarks that could similarly trigger ATR activation in human cells and shows that phospho-H2A and Brc1 are a critical part of the network that maintains genome integrity during DNA replication.

## Introduction

During DNA replication cells are particularly vulnerable to loss of genetic information and mutation [1]. The DNA replication checkpoint pathway monitors the genome to detect and stabilize stalled forks, initiate repair, and delay mitotic entry until DNA damage is repaired [2]. Checkpoint activation during replication is triggered by the kinase ATR, which is crucial for maintenance of genome stability during S-phase [3]. Chromosomal instability, neuronal defects, and premature aging characterize human Seckel syndrome caused by ATR deficiency, whilst corresponding mutants of *Saccharomyces cerevisiae* Mec1 or *Schizosaccharomyces pombe* Rad3 display chromosomal instability and are hypersensitive to a wide spectrum of genotoxins [3]. ATR orthologs are important for maintaining genome integrity in the absence of genotoxins, yet little is known about the endogenous sources of ATR activation. Candidates include highly repetitive DNA, natural replication fork barriers (RFBs), and chromosomal “fragile sites”, which may present obstacles to replication [1], [4], [5]. However, it is unknown whether specific chromosomal domains are responsible for Rad3 activation during an unperturbed cell cycle.

One of the most rapid and highly conserved responses to DNA damage is phosphorylation of histone H2AX or histone H2A in yeast [6]–[8]. ATR^Mec1/Rad3^ and the related kinase ATM^Tel1^ catalyze this phosphorylation [6]–[8]. Phospho-H2AX (γH2AX) spreads over extensive chromatin domains flanking DNA double-strand breaks (DSBs) [9], [10]. Mice deficient for γH2AX are immunocompromised, display sensitivity to ionizing radiation (IR), and moderate genomic instability, while yeast with H2A mutations that abolish phosphorylation are moderately sensitive to a variety of DNA damaging agents [7], [8], [11], [12]. Although typically associated with DSBs, γH2AX also forms in response to genotoxic stresses that stall or collapse replication forks [13]–[18]. The role of γH2AX in repair of replication-associated damage is largely unexplored. γH2AX may have a direct function in stabilization of stalled forks [15], [18] and has been linked to the Fanconi Anemia/BRCA pathway for resolving stalled forks [17].

The best-characterized γH2AX-binding proteins are mammalian Mdc1 and fission yeast Crb2, which associate with γH2AX through their C-terminal tandem BRCT domains [19], [20]. We recently discovered that Brc1 is a second γH2A-binding protein in fission yeast [21]. Like Mdc1 and Crb2, Brc1 uses a pair of C-terminal BRCT domains to bind γH2A. However, unlike Mdc1 or Crb2, which play critical roles in the checkpoint responses to DSBs, Brc1 is specifically required for repair of replication-associated DNA damage [22], [23]. Indeed, Brc1 mutants that cannot bind γH2A are sensitive to replication-specific genotoxins, as are fission yeast “*htaAQ*” mutants lacking the phosphorylation site in histone H2A [8], [21]. Brc1 is structurally related to budding yeast Rtt107/Esc4 and mammalian PTIP [23]–[25]; however, the mechanisms by which these proteins protect genome integrity remains a mystery.

Rad3, γH2A and Brc1-defective mutants all have increased frequencies of spontaneous Rad22^Rad52^ foci during S-phase, indicating homologous recombination (HR) repair of stalled or collapsed replication forks [8], [26]. This phenotype is shared with mutants lacking Rqh1, a RecQ-like DNA helicase that is required for genome stability during S-phase and which has critical functions in maintaining the copy number of the repeated ribosomal RNA gene (rDNA) loci [27]–[30]. These phenotypes may be partially explained by endogenous DNA damage arising from normal metabolic processes, but other factors could be specific fragile sites, where DNA-bound protein complexes, transcriptional machinery, or other uncharacterized chromatin structures interfere with DNA replication. To address whether Rad3 is activated at specific chromosomal domains during replication, we mapped γH2A formation during an unperturbed S-phase using whole-genome microarrays. These studies, which by proxy reveal the site of action of ATR^Rad3^ or ATM^Tel1^, show that γH2A decorates a surprisingly diverse array of chromosomal structures, including all heterochromatin domains. There are interesting similarities and differences with the genome-wide distribution of γH2A in budding yeast [31]. We also demonstrate that Brc1 binding to γH2A is critical in the absence of Rqh1, indicating a crucial role for the Rad3-γH2A-Brc1 pathway in maintaining genome integrity during S-phase.

## Results

### Genome-wide map of γH2A during DNA replication in *S. pombe*

To address whether specific chromosomal domains trigger DNA damage responses in the absence of exogenous genotoxins, we wanted to map the distribution of γH2A in the *S. pombe* genome using chromatin immunoprecipitation and tiled microarray (ChIP-on-chip) analysis. Since γH2A ChIP-on-chip analysis has not been performed in *S. pombe*, we first tested if γH2A could be detected at a site-specific DNA DSB made by activating expression of the HO-endonuclease [32]. γH2A ChIP was performed with a phospho-H2A specific antibody [33]. As a control we used a strain in which both histone H2A genes (*hta1* and *hta2)* encode proteins that cannot be phosphorylated, hereafter referred to as the *htaAQ* mutant [8]. Upon HO-endonuclease induction, γH2A was detected in a broad domain spanning more than 40 kb surrounding the DSB, but was reduced within a 2 kb region around the break site, which is similar to previous observations in *S. cerevisiae* [34] (Figure S1).

**Figure 1:**
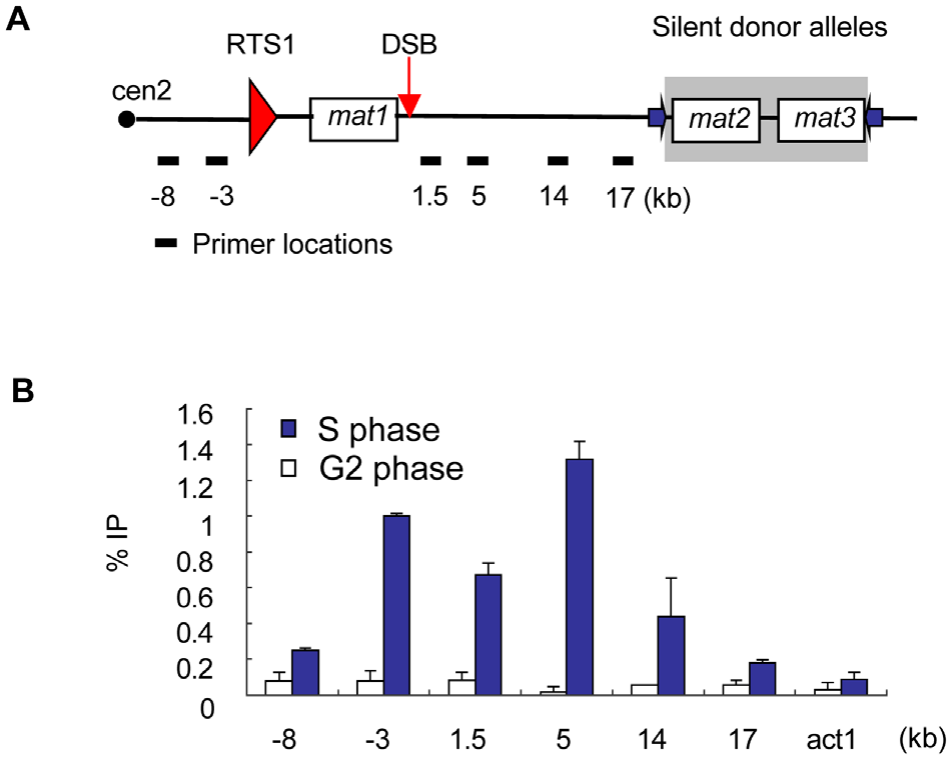
γH2A forms in the mating-type locus during DNA replication. (A) Diagram of a typical *S. pombe* mating-type (MT) locus (not to scale). Symbols: cen2 –centromere2, RTS1-polar replication fork barrier, DSB- imprinting site where DSB forms during replication; purple arrows - inverted repeats. Primer names correspond to distance in kilobases from the DSB. (B) ChIP-qPCR analysis of γH2A distribution at the MT locus in G2 and S phase. Cells were synchronized in G2 phase using the *cdc25-22* allele and S-phase progression was monitored using septation index. ChIP enrichment at the indicated sites was quantitated %IP.

Next, to obtain a positive control site for the ChIP-on-chip experiments, we examined if γH2A can be detected at the only known fragile site in *S. pombe*, located in the mating-type (MT) locus. Mating-type switching involves DSB formation and recombination, but it does not elicit a checkpoint arrest, as evidenced by normal cell-cycle progression in switching strains. The configuration of the MT locus varies with strain mating type, so a typical MT locus of a standard *h^90^* homothallic strain is shown in Figure 1A. The *mat1* gene is actively expressed and determines the mating-type, which is either *h-* or *h+. Mat1* is replicated from the right side, which induces a DSB at a fragile site next to *mat1* [35]–[37]. A polar replication fork barrier (RFB), called RTS1, blocks replication forks from the centromere. The induced DSB initiates recombination with inactive donor alleles, *mat2* (*h+*) or *mat3* (*h−*), located in a heterochromatic domain, to switch the mating-type at *mat1* [37]. We checked γH2A formation at the *mat1* DSB in a “Donorless” strain, which lacks the donor alleles, and uses sister-chromatid recombination to repair the break [36], [38]. Since DSB formation at *mat1* is transient and occurs during replication, ChIP analysis was performed on cells that were enriched in S-phase by using the “*cdc25-22* block and release” protocol to synchronize cell cycle progression, as described in Materials and Methods [39], [40]. We observed that γH2A was highly enriched near the DSB in the MT locus in S-phase, but not in G2 (Figure 1B). Typical *h−* and *h+* laboratory strains also contained γH2A in the MT locus (data not shown). This demonstrated that γH2A is triggered by a transient DSB during mating-type switching and therefore the MT locus should be a reliable positive control in γH2A ChIP-on-chip analysis.

**Figure 2:**
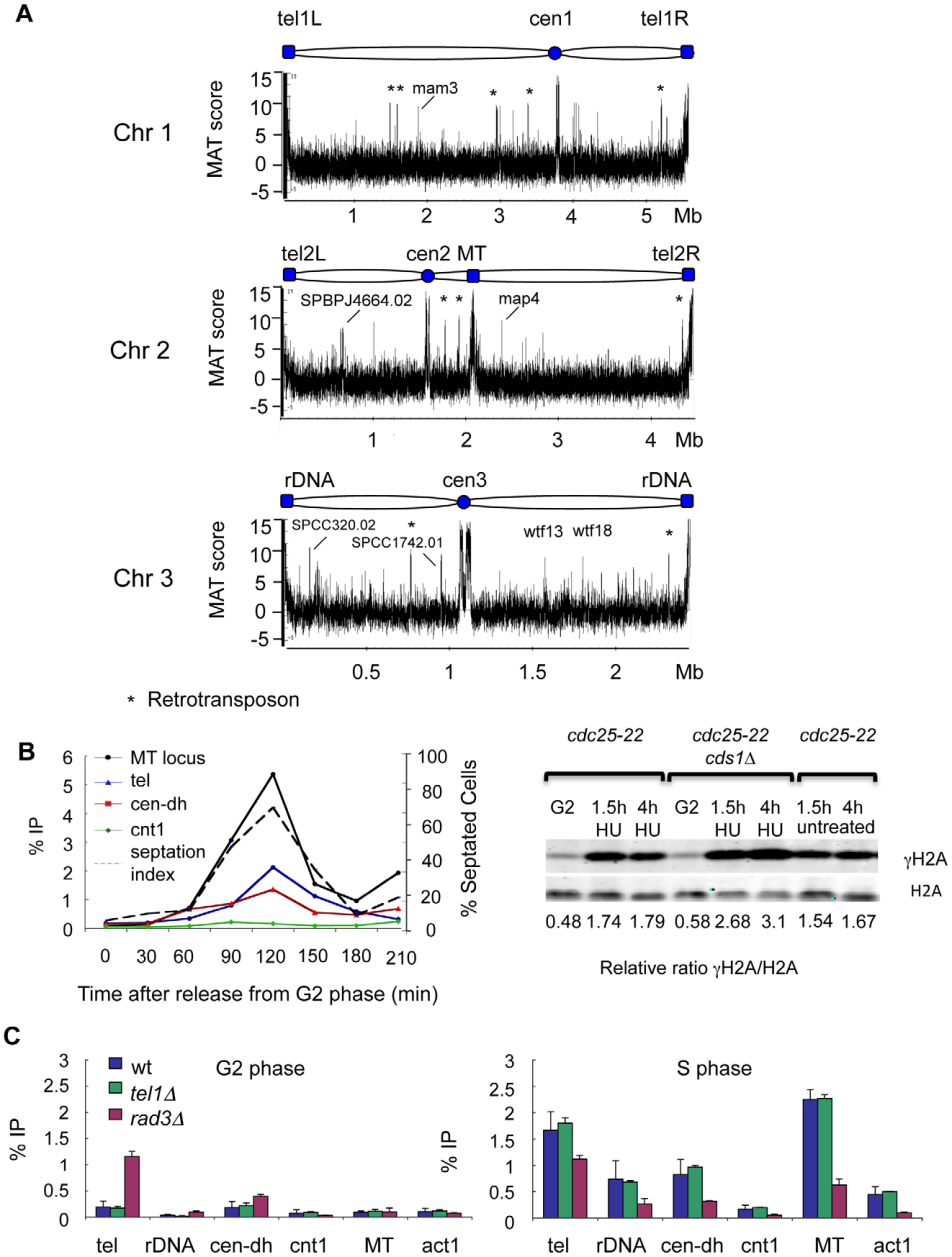
Genome-wide localization of γH2A during DNA replication. (A) Genome-wide (ChIP-on-chip) distribution of γH2A during S phase. Schematics represent the three *S. pombe* chromosomes with key structural features (top). Tel- telomere; cen-centromere; MT-mating-type locus; rDNA-ribosomal DNA. Enrichment of γH2A is displayed as MAT scores (y-axis). Chromosome coordinates (x-axis, in megabases, (Mb)) downloaded from the *S.pombe* Genome Project (Sanger Center: www.sanger.ac.uk/Projects/S_pombe). (B) (left) γH2A formation occurs specifically during S phase. ChIP-qPCR timecourse analysis of γH2A enrichment at the indicated sites was performed by synchronizing cells using *cdc25-22* block and release, and ChIP samples were collected every 30 min. Cell cycle progression was monitored by septation index. (right) Western blots comparing levels of γH2A in *cdc25-22* wild type (Cds1+) and *cdc25-22 cds1Δ* cells released into 12 mM HU from G2 arrest. As an untreated control *cdc25-22* wild type cells were released from G2 in the absence of HU. (C) Rad3 is the main kinase that phosphorylates H2A during unperturbed S phase. γH2AChIP-qPCR in wild type, *rad3Δ*, or *tel1Δ* cells synchronized as in (B), samples were collected in G2 and S phase. Primers: MT–5 kb from DSB in MT-locus; tel-subtelomere 1; cen-dh – centromere dh repeats; cnt – centromere 1 core; rDNA- 35S ribosomal DNA gene.

The “Donorless” strain was used for genome-wide localization analysis of γH2A. This MT locus configuration has no adverse effects on viability [38]. Samples were collected from cultures synchronized in S-phase using *cdc25-22* block and release. DNA was hybridized to whole-genome tiling microarrays with 20 bp resolution and the data was analyzed using the Model-based Analysis of Tiling Array (MAT) algorithm [41]. ChIP input DNA was used as a control for the microarray data analysis. Predicted γH2A enrichment at each site is displayed as “MAT score” as explained in the supporting information (Text S1). Note that MAT score is a measure of probability and is not a quantitative measure of protein amount. Approximately 400 statistically validated peaks were identified, all of which had MAT scores of 5 or higher. The resulting genome-wide landscape of γH2A encompassed a diverse array of genomic features, including the mating-type locus, the rDNA loci, and surprisingly all heterochromatin regions, including the centromeres and telomeres (Figure 2A). γH2A formation was also observed at mobile genetic elements (Tf2-type retrotransposons and *wtf* elements) and in a subset of gene coding sequences (Table S1).

To confirm γH2A enrichment at the identified loci and determine if the phosphorylation was S-phase specific, we monitored γH2A levels by conventional ChIP during the cell cycle. Cells were synchronized in G2 phase using *cdc25-22* temperature arrest, and ChIP samples were collected every 30 min after return to permissive temperature. Cell cycle progression was assessed by monitoring the septation index. Quantitative PCR (qPCR) primers were designed in regions predicted to have high γH2A levels by ChIP-on-chip analysis. Our results showed that γH2A enrichment in all regions was low in G2, increased and peaked in S-phase (coinciding with the rise in septation index), and decreased as the cells reentered G2 (Figure 2B). The highest level of enrichment was detected in the MT locus (MT). The telomeres (tel) had intermediate γH2A levels, and lower signals were detected at the outer centromere repeats (cen-dh). As predicted by ChIP-on-chip analysis, no enrichment was detected in the centromere core (cnt1). Transient formation of γH2A during replication suggests that these genomic regions are especially susceptible to replication fork arrest or collapse during S-phase.

These data indicated that generation of γH2A is a normal event in S-phase. To gauge the level of γH2A relative to that caused by replication fork arrest or collapse, we released *cds1* ^+^ or *cds1Δ* cells from the *cdc25-22* arrest into media containing 12 mM hydroxyurea (HU). The amount of γH2A in wild type cells treated with HU was comparable to untreated cells harvested in S-phase, whereas replication fork collapse caused by HU treatment of *cds1Δ* cells led to substantially higher γH2A levels (Figure 2C).

Next, we investigated the relative contributions of Rad3 and Tel1 checkpoint kinases to γH2A formation during unperturbed replication. Using the *cdc25-22* arrest and release protocol to enrich cells in S-phase, we observed a large decrease in γH2A at every site in the absence of Rad3, while *tel1Δ* mutants were similar to wild type (Figure 2C). The effect of the *rad3Δ* mutation was weakest at the telomere, indicating a major role for Tel1 at telomeres, at least in the absence of Rad3. Indeed, Rad3-defective cells have short telomeres and *rad3*Δ *tel1Δ* double mutants completely lose telomeres [42], [43]. These facts are consistent with our data showing a high γH2A signal at the telomeres in G2-phase *rad3Δ* cells (Figure 2C). As expected, deletion of both kinases completely abolished γH2A formation (Figure S2).

### γH2A at the mating-type locus

**Figure 3:**
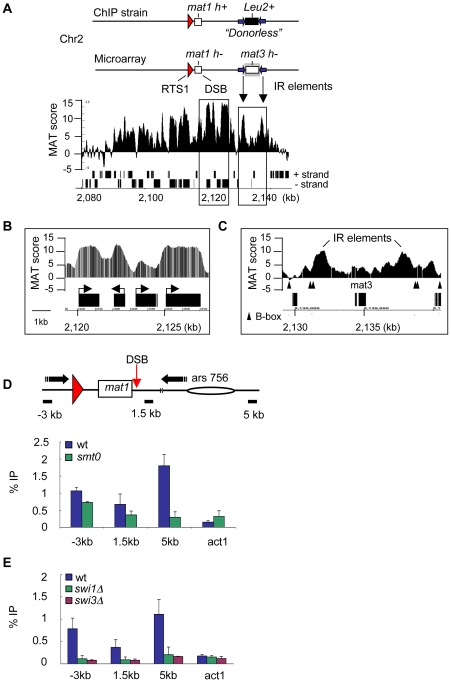
The RTS1 fork barrier at the MT locus triggers γH2A formation. (A) Detailed landscape of γH2A in the MT locus as determined by ChIP-on-chip analysis in Figure 2A. The top diagram compares the MT locus configuration of the Donorless ChIP strain to sequences present on the Affymetrix *S. pombe* Tiling 1.0 FR microarray. Locations of key features correspond to the ChIP-on-chip data coordinates shown below. Both strains contain the RTS1 barrier. The ChIP strain is mating-type *h+* at *mat1*, and lacks the silent donor alleles, which were replaced with a Leu2 marker. The microarray strain is mating-type *h-* at *mat1* and contains only the *mat3-h-* donor allele. Both strains contain inverted repeat (IR) elements flanking the Leu2 or *m*at3 cassettes (purple arrows). Black rectangles below plot represent genes. Black boxes correspond to magnified regions shown in (B) and (C). (B) An example of preferential enrichment of γH2A in gene coding regions. (C) Detailed examination of γH2A at the IR boundary elements shows that γH2A spreading is restricted by B-box sequences (black triangles). (D) The RTS1 fork barrier leads to γH2A in the absence of the DSB. γH2A ChIP was performed in wild type and *smt0* strains synchronized by *cdc25-22* block. Diagram shows qPCR primer locations (black rectangles) relative to the DSB at Mat1. (E) γH2A formation at the MT-locus depends on Swi1-Swi3. γH2A ChIP was performed in the indicated strains as in (D) The locations of qPCR primers are indicated in diagram in (D).

The MT locus showed the highest γH2A levels on the array (Figure 2A). Detailed analysis revealed a broad, non-uniform distribution of γH2A spanning approximately 50 kb around the *mat1* DSB and the RTS1 barrier, with a trough immediately adjacent to the DSB and enrichment peaking within 10 kb of the DSB (Figure 3A). The diagrams above the plot correlate locations of MT locus features to the microarray data and compare the microarray MT locus configuration to the Donorless strain. The main difference is at *mat1*, due to different mating types, and in the silent region, where a LEU2 marker replaced the *mat3* allele in the Donorless strain. The silent region in both strains is flanked by inverted repeats (IR). Closer inspection revealed additional interesting features in the γH2A pattern. First, H2A phosphorylation was preferentially distributed in gene coding regions, an example of which is shown in Figure 3B. This trend was observed genome-wide and could be due to lower nucleosome occupancy in highly AT-rich *S. pombe* intergenic regions [44]. Second, we identified two γH2A peaks at the IR elements flanking the missing *mat3* site, which has a gap in γH2A signal (Figure 3C). These IRs act as boundary elements that prevent the spread of heterochromatin out of the MT silent region [45]. A comparison to histone modifications in this region revealed that γH2A colocalized with H3 K9 methylation, a marker of heterochromatin, which peaks at the IRs [45]. The specific boundary elements are B-box sequences bound by TFIIIC, a factor associated with RNA polymerase III [45]. We found that γH2A peaks are bounded by B-box sequences, similar to heterochromatin (Figure 3C). There were no additional boundaries to γH2A spreading in the MT locus, and the overall signal attenuated as distance from the DSB increased.

### γH2A formation at the RTS1 replication fork barrier

We next tested whether the RTS1 RFB alone was sufficient for triggering γH2A formation by using a strain called “*smt0*”, which contains the RTS1 barrier but lacks the DSB imprinting site [46]. We detected γH2A on the left side of RTS1 where the replication fork stalls at the barrier, but the signal greatly decreased on the right side of the DSB (Figure 3D). Thus, although the majority of γH2A at the MT locus was due to the DSB, replication fork pausing at RTS1 also triggers γH2A formation.

Both RTS1 barrier activity and DSB formation depend on the Swi1-Swi3 complex, which travels with the replisome and mediates replication fork pausing at natural barriers [47], [48]. To confirm that H2A phosphorylation was dependent on RFB activity, γH2A ChIP was performed in *swi1Δ* or *swi3Δ* strains that contain RTS1 and the DSB site. The formation of γH2A at the MT locus was eliminated in both *swi1Δ* and *swi3Δ* cells (Figure 3E). These data demonstrate that both a transient DSB and a natural replication fork barrier trigger γH2A, which depends on replication fork pausing mediated by the Swi1-Swi3 complex.

**Figure 4:**
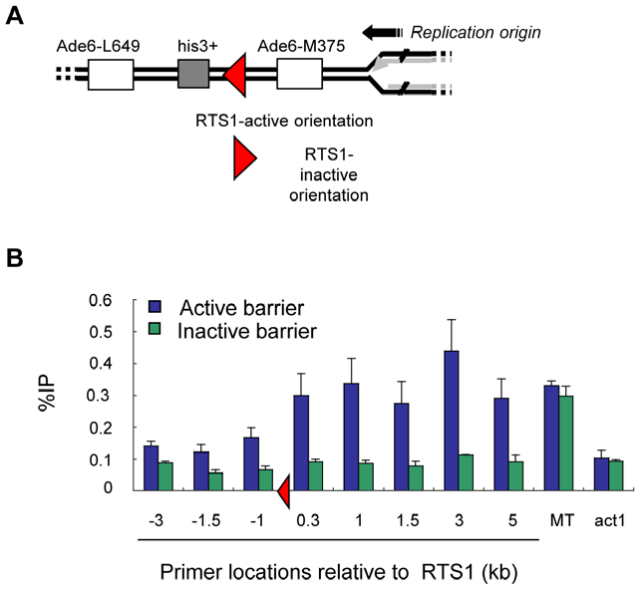
γH2A forms at an ectopic RTS1 replication fork barrier. (A) Diagram illustrates position of RTS1 barrier located between two direct repeats of Ade6 alleles. Diagram based on [49]. (B) γH2A ChIP at sites surrounding the active or inactive RTS1 fork barrier. ChIP was performed on asynchronous cultures. A primer –3 kb from the RTS1 barrier in the MT locus was used as positive control.

We next examined whether γH2A can form at an ectopic RTS1 fork barrier. We used a strain where RTS1 was inserted in the Ade6 locus, between two direct repeats (Figure 4A) [49]. This is a strong polar replication fork barrier and outside the MT locus fork stalling at RTS1 promotes recombination, which occurs without fork breakage, DSB formation, or checkpoint activation [49]. As a control we used an “inactive” strain in which the barrier is oriented in the opposite direction, thereby avoiding fork arrest (Figure 4A). ChIP analysis revealed a strong asymmetric enrichment of γH2A up to 5 kb away from the active RTS1 barrier (Figure 4B). The majority of γH2A was located on the right side of the barrier, where fork stalling occurs and recombination is initiated [49]. There was comparatively little γH2A on the left side of the barrier and no change in γH2A levels was detected when RTS1 orientation was reversed. A primer near RTS1 in the MT locus was used as a positive control for the experiment and showed similar levels of γH2A in both strains. These data show that fork stalling at a polar fork barrier triggers formation of an asymmetric γH2A domain and suggests that γH2A may mark recombination hotspots in the genome.

### γH2A is associated with fork barriers in the rDNA

**Figure 5:**
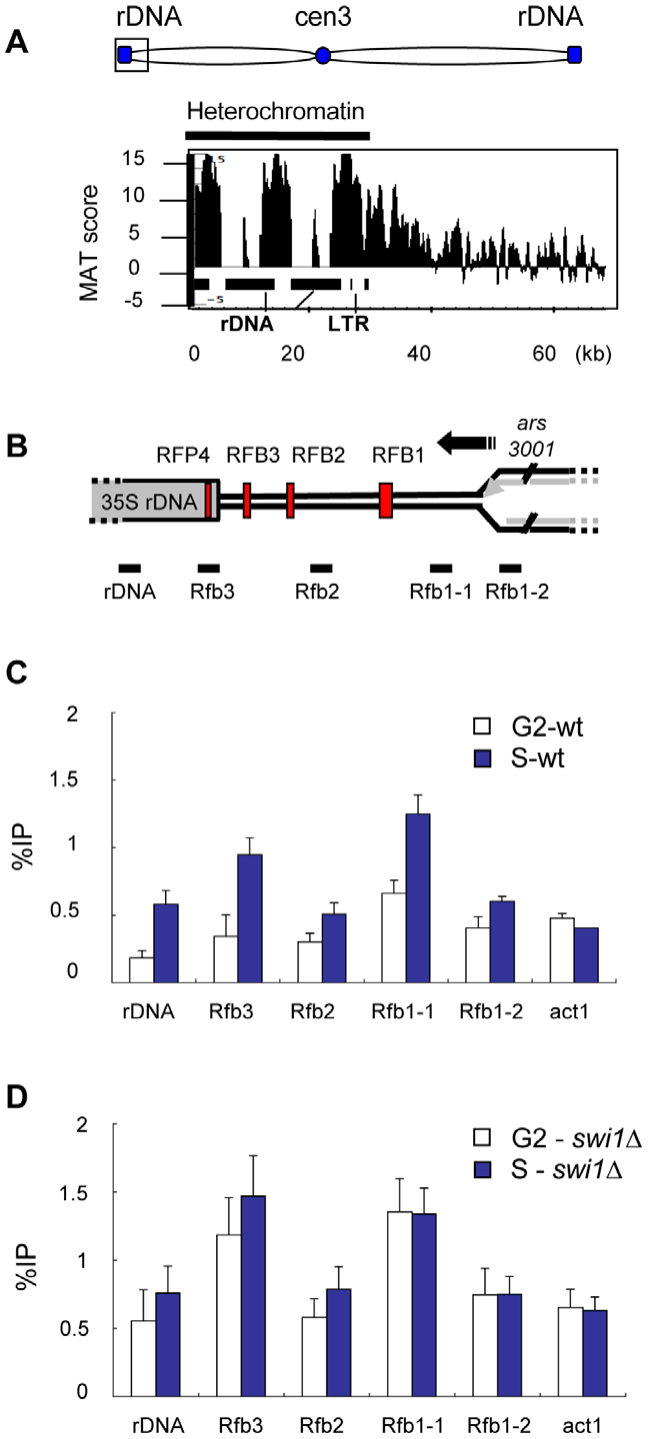
γH2A is highly enriched in the rDNA repeats during S phase. (A) Detailed ChIP-on-chip distribution of γH2A in the rDNA on the left arm of chromosome 3. Black rectangles (below graph) represent 35S rDNA gene repeats. (B) Diagram of one rDNA repeat (not to scale) shows the location of the four replication fork barriers (red vertical bars) relative to the 35S rDNA genes, the direction of replication (black arrow) from the *ars3001* replication origin, and qPCR primer locations, below graph. (C, D) γH2A ChIP at the rDNA was performed in wild type and *swi1Δ* strains synchronized by *cdc25-22* block and analyzed by qPCR with the indicated primers.

Multiple RFBs are present in the rDNA loci, which are located on the subtelomeric arms of chromosome 3 [50], [51]. A large γH2A domain was detected in the rDNA, which decreased to background levels within 10 kb outside this region (Figure 5A). The rDNA is organized into ∼150 tandem repeats, but the microarray probes represented only a few repeat units, as shown below the γH2A plot. The gaps between γH2A peaks reflect the absence of microarray probes at repetitive DNA sequences. Because rDNA sequences were poorly represented on the microarray we further examined the γH2A distribution using conventional ChIP analysis. A diagram of a single rDNA repeat is shown in Figure 5B. Each repeat consists of the 35S rDNA genes, a replication origin (*ars3001*), and four distinct replication fork barriers (RFB1–3 and RFP4). The rDNA is highly transcribed, and the RFBs between the repeats facilitate unidirectional replication to prevent head-on collisions between the replisome and transcription complexes [52]. Replication fork pausing at rDNA barriers also regulates recombination, which is necessary for dynamic maintenance of rDNA copy number [52], [53]. Using the indicated primers (Figure 5B), we detected γH2A enrichment throughout the rDNA locus, including the gene coding sequences and surrounding all four RFBs (Figure 5C), as predicted by the ChIP-on-chip analysis. Enrichment was detected only in S-phase and not in G2-arrested cells, which suggests that RFB activity triggers γH2A formation. Interestingly, γH2A levels at the barriers correlated with barrier strength, with RFB1 being the strongest barrier, and RFB2 the weakest [51].

The Swi1-Swi3 complex is required for replication fork stalling at the three intergenic RFBs (RFB1-3) [51], [54]. The RFP4 barrier is atypical, and is thought to be caused by collisions between transcription and replication [51]. Deletion of Swi1 exacerbates fork stalling at RFP4 [51]. ChIP analysis in *swi1Δ* cells revealed high levels of γH2A in the rDNA in both G2 and S-phase, which contrasts the S-phase-specific γH2A formation in wild type cells (Figure 5D). The high levels of γH2A could be caused by fork collapse and/or recombination due to replisome collisions with transcription at RFP4. Increased recombination-associated Rad22 foci were observed in *swi1Δ* mutants, which supports these conclusions [48], [55]. Overall, these results suggest that the Rad3-γH2A pathway has a role in the maintenance of rDNA during normal replication, and that perturbation of RFB activity increases genomic instability in the rDNA locus.

### γH2A formation near tDNA barriers

**Figure 6:**
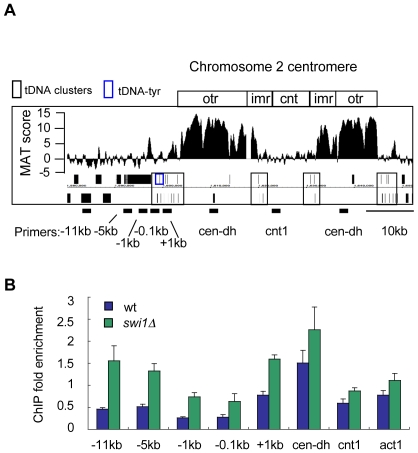
γH2A accumulates near tDNAs in the absence of Swi1. (A) Detailed ChIP-on-chip distribution of γH2A near tDNA clusters bordering centromere 2. Boxed diagram above plot shows locations of centromere features: otr- outer repeats, imr- inner repeats, cnt – centromere core. Black boxes below plot represent genes. Location of qPCR primers are shown below graph and name correspond to distance from tDNA^TYR^ (B) ChIP-qPCR of γH2A near tDNA^TYR^ in wild type and *swi1Δ* cells.

Replication fork pausing can also occur at the highly transcribed tDNA genes [56], [57]. However, we did not detect γH2A near tDNAs in our genome-wide analysis, with the exception of the centromeres, where clusters of tDNAs surround a γH2A domain located within the heterochromatic centromeric repeats (Figure 6A). These tDNA clusters serve as boundary elements that prevent heterochromatin spreading from the centromere repeats [58]. In a similar manner, the tDNA clusters seem to prevent γH2A spreading from the repeats, because beyond the tDNAs the γH2A MAT scores drop sharply (Figure 6A). The centromeric γH2A signal could be caused by replication through heterochromatin, which is discussed in the next section.

There are some differences between the structure of RTS1 and rDNA RFBs and the tDNAs barriers. The tDNA barriers are nonpolar and weaker than RTS1, and fork stalling at tDNAs does not depend on Swi1 [56]. However, Swi1 has a general role in facilitating replisome progression through tDNAs and the absence of Swi1 increases recombination at tDNAs, likely from increased collisions between the replisome and transcription [56]. We analyzed the effect of Swi1 deletion on γH2A levels near the leftmost tDNA cluster bordering centromere 2 (Figure 6A). Primers were designed around the tDNA^Tyr^ gene. ChIP in wild type cells showed no appreciable γH2A enrichment upstream of the tDNA^Tyr^ (Figure 6B). However, in *swi1Δ* mutants we detected increased γH2A levels around the tDNA cluster, as far as 11 kb away. Our data suggest that Swi1 is needed to promote replication fork progression through the tDNAs in a manner that avoids triggering γH2A formation.

### γH2A forms in fission yeast heterochromatin regions

**Figure 7:**
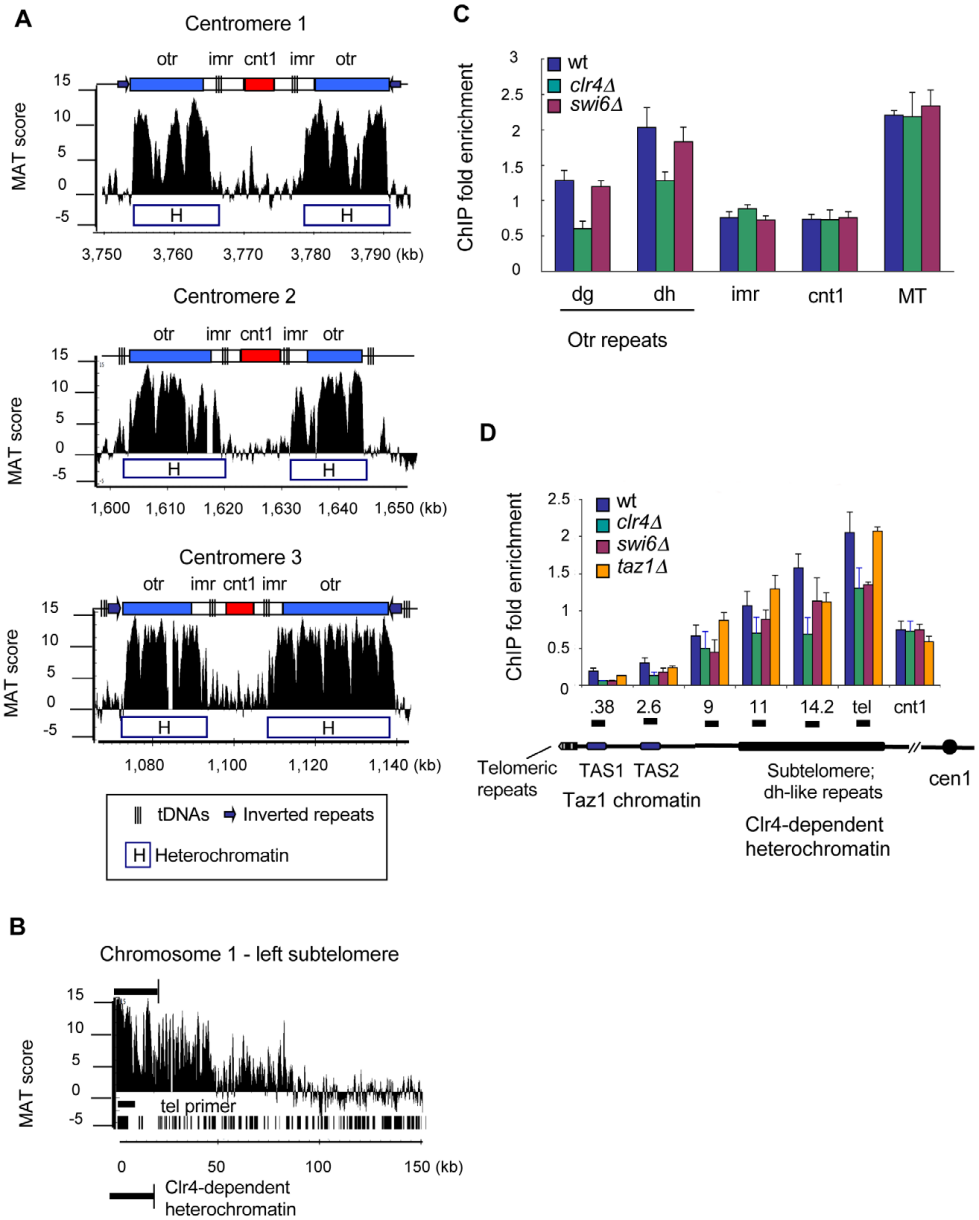
γH2A in the centromeres and telomeres is associated with Clr4-dependent heterochromatin. (A) Detailed ChIP-on-chip distribution of γH2A at the centromeres. Diagrams above each plot indicate key centromere features: otr – outer centromere dg/dh repeats; imr- inner repeats; cnt –centromere core. (B) Detailed ChIP-on-chip distribution of γH2A in the subtelomere of Chromosome 1. Location of genes and “tel” qPCR primer is shown as black bars below plot. (C) γH2A levels were reduced at the centromeres in *clr4Δ* cells. γH2A ChIP was performed in indicated strains synchronized by *cdc25-22* block. The dg and dh primers are located in the outer centromere repeats. ChIP data is shown as Fold Enrichment, which was calculated relative to the act1 gene. (D) γH2A domain in the subtelomeres colocalizes with heterochromatin and decreases in *clr4Δ* and *swi6Δ* cells. ChIP was performed as in (C). Diagram below graph shows the organizational structure of the telomeres (not to scale) and was based on [70]. Primer locations are marked by horizontal black bars (top) and correspond to distance in kilobases from the telomeric repeats.

Interestingly, our genome-wide analysis revealed prominent γH2A peaks in all heterochromatic loci, including centromeres, telomeres, silent MT locus, and rDNA (Figure 2A). Heterochromatin is established during S-phase, and is initiated by methylation of histone H3 on lysine 9 (H3K9me) by the Clr4 methyltransferase, followed by binding of Swi6, a homolog of HP-1, which regulates heterochromatin spreading and function [59]. Additionally, tandem repeat sequences called “*dg/dh* repeats”, located in the outer centromeres, subtelomeres, and the silent MT region, are transcribed into small interfering RNA (siRNA) to promote heterochromatin formation during S-phase [60]. Fission yeast centromeres consist of a central core (*cen*), surrounded by inner (*imr)* and outer (*otr*) regions [59]. The *otr* contains *dg/dh* repeats and is the site of Clr4-dependent heterochromatin assembly. Detailed analysis revealed that γH2A was enriched in the *otr* and a part of the *imr* at all three centromeres (Figure 7A). Remarkably, this pattern almost perfectly matches the distribution of heterochromatin markers Swi6 and H3K9me [61]. The spreading of γH2A beyond heterochromatin was blocked by tDNA clusters and inverted repeat (IR) boundary elements, which prevent heterochromatin spreading [58], [61]. As discussed earlier, γH2A in the MT locus silent region was similarly restricted by IR boundary elements. The paucity of γH2A in the centromere core can also be attributed to the reduced density of H2A/H2B dimers from this part of the centromere [62], [63]. It is intriguing that Rad3-dependent phosphorylation of H2A may be inhibited by the same boundary elements that prevent the spread of heterochromatic epigenetic modifications.

The telomere sequence coverage on the *S.pombe* microarray is incomplete and the best coverage is of the subtelomere located on the left arm of chromosome 1 [61]. Detailed analysis showed that a large non-uniform γH2A domain extended up to 50kb away from the subtelomere (Figure 7B). Like the centromere *otr* repeats, the subtelomere is modified by H3K9me and Swi6, and contains *dg/dh*-like elements that are transcribed into siRNAs [59]. Comparison of the subtelomeric γH2A pattern to H3K9me revealed that γH2A colocalized with heterochromatin, but unlike at the centromeres, the signal spread more than 50 kb beyond the end of H3K9me chromatin [61]. This region of the subtelomere is also transcriptionally repressed, but via Clr6/Clr3 histone deacetylases, independently of Clr4 methylation [64], [65]. It contains a significant number of meiotic genes, which are expressed during nitrogen starvation [66]. Thus, γH2A formation in subtelomeric regions is associated with two different types of repressed chromatin. The subtelomeres of chromosome 3 contain rDNA repeats, which also have H3K9 methylated heterochromatin [61]. Heterochromatin in the rDNA is limited from spreading by long terminal repeats (LTRs)[61], however, γH2A enrichment continued past these boundaries (Figure 5A). γH2A was also present at Tf2-type retrotransposons and *S. pombe-*specific *wtf* elements, which are transcriptionally repressed by Clr3/Clr6-mediated histone deacetylation [64]. The distribution of γH2A at Tf2s and *wtfs* was confined by LTRs (Figure S3). Due to high sequence similarity among the members of transposon families [67] it was not possible to distinguish whether all Tf2s and *wtf* elements, or only a sub-population, were enriched in γH2A. Functionally, the role of *wtf* elements is unknown, but they are very highly expressed during meiosis [66], [67]. Overall, the association of γH2A with repressed chromatin during S-phase suggests that heterochromatin may impede replication fork progression.

### γH2A-containing genes

Gene coding regions enriched with γH2A were distributed on all three chromosomes (Table S1) As mentioned earlier, the majority of these genes are located in subtelomeric regions, in silenced chromatin mediated by Clr6/Clr3 histone deacetylases (Figure 7B). Analysis of gene ontology (GO) terms and expression data in the *S. pombe* GeneDB database (www.genedb.org/genedb/pombe) revealed that many of the γH2A enriched genes are involved in mating and meiosis or are upregulated in response to environmental stress (Table S1). The largest group of genes represented was cell adhesion proteins many of which are needed during mating and share a common structure in the form of internal tandem repeats [68]. An example of this is the SPBPJ4664.02 gene locus, which is predicted to code for a protein with ∼250 copies of 12-amino acid repeats according to the S. pombe GeneDB database (www.genedb.org/genedb/pombe) (Figure S3). Thus, our data suggests that induction of γH2A in genes could be triggered by either repressed chromatin or repetitive DNA sequences.

### γH2A at centromeres and subtelomeres is associated with establishment of heterochromatin

The prominent enrichment of γH2A at heterochromatic loci suggested that a relationship exists between heterochromatin formation and γH2A during DNA replication. On one hand, heterochromatin contains many features that could impede replisome progression, including repetitive sequences, compacted higher-order DNA structures, and multiple DNA-bound proteins [4], [69]. On the other hand, the striking similarity between heterochromatin distribution and γH2A prompted us to examine if cells lacking γH2A (*htaAQ* mutant) had defects in heterochromatin function. We tested chromosome segregation defects, loss of gene silencing, sensitivity to microtubule inhibitors, and telomere length alterations, but did not detect any defects (data not shown). A crucial function of heterochromatin is recruitment of cohesin, and high levels of the cohesin subunit Rad21 are present at the centromere *dg/dh* repeats and in the subtelomeres [70], [71]. γH2A has been shown to recruit cohesin to sites of induced DSBs [72], [73]. However, ChIP analysis of Rad21 binding at the centromeres and subtelomeres did not show significant differences between wild type cells and *htaAQ* mutants (Figure S4). These results led us to conclude that γH2A either does not participate in heterochromatin function, or it is part of a redundant pathway, which masks any defects.

We next tested whether γH2A formation was dependent on the presence of heterochromatin by examining γH2A levels in the absence of factors that mediate heterochromatin formation. ChIP of γH2A in *clr4*Δ and *swi6Δ* mutants in S-phase showed that deletion of Clr4 but not Swi6 led to decreased γH2A signals in the centromere *dg/dh* repeats (Figure 7C). This suggests that Clr4-dependent heterochromatin formation triggers γH2A formation in the centromeres during replication. The lack of an effect in *swi6Δ* mutants indicates that a barrier to replication is still present in the centromeres. This idea is supported by studies of heterochromatin replication timing, which showed that centromere replication is partially impeded by Clr4-dependent heterochromatin in *swi6Δ* mutants [74].

Next, we analyzed γH2A at the telomeres in *clr4Δ*, *swi6Δ*, and *taz1Δ* mutants. Taz1 is a telomere end-capping protein that facilitates replication through the telomeres and regulates telomere homeostasis [75], [76]. Since the microarray probe coverage was limited to the subtelomere, we extended our analysis of γH2A further into the telomeres. The subtelomere region is followed by telomere-associated sequence (TAS) elements, and then tandem telomeric repeats (bottom diagram, Figure 7D). We observed high γH2A enrichment in the subtelomere, as predicted by the ChIP-chip analysis, but the signal rapidly decreased in the TAS region (Figure 7D). The subtelomere contains Clr4-dependent heterochromatin, whereas the telomeric repeats bind Taz1 [61], [70], [77]. The distribution of γH2A was limited to the subtelomere region similar to H3K9me and Swi6 binding [70], [77]. As a positive control for the ChIP experiments we confirmed that Taz1 binds in the TAS and the telomere repeats and not in the subtelomere (Figure S5). The subtelomeric γH2A signal decreased in both *clr4Δ* and *swi6Δ* mutants, but not in *taz1*Δ mutants (Figure 7D). These data demonstrate that γH2A formation in the subtelomeres is linked to the presence of heterochromatin established by H3K9 methylation and Swi6 binding and is independent of Taz1. Overall, our results indicate that transient γH2A formation in the centromeres and subtelomeres during DNA replication is associated with the presence of Clr4-dependent heterochromatin and partially depends on Swi6.

### γH2A mediates Brc1 recruitment to heterochromatic loci

**Figure 8:**
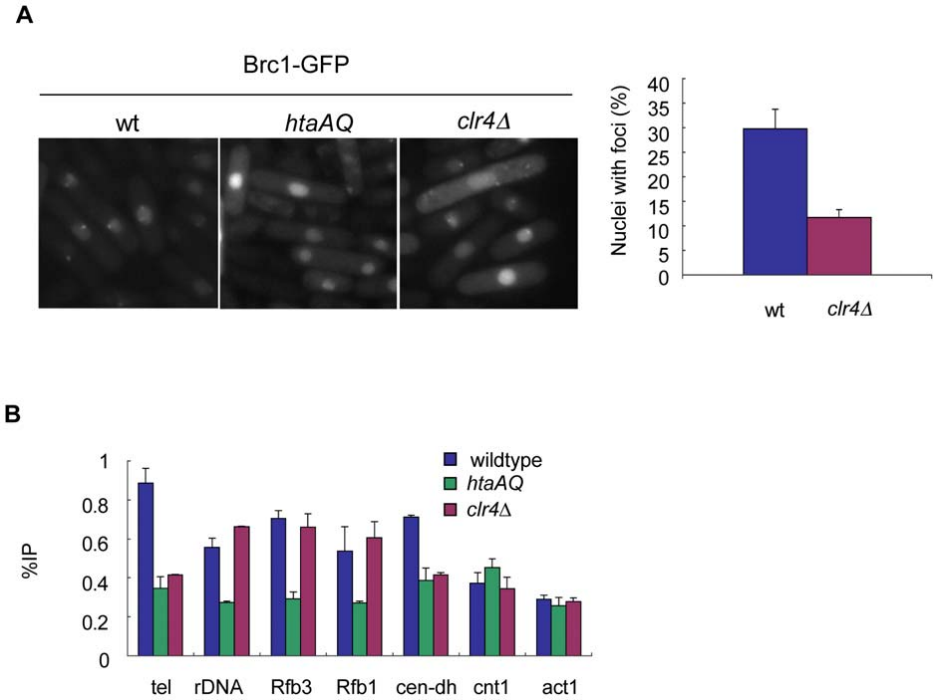
Brc1 recruitment to γH2A sites is partially dependent on Clr4. (A) Spontaneous Brc1-GFP foci are reduced in *clr4Δ* mutants. Live cell microscopy of Brc1-GFP overexpressed using the *nmt* promoter in wild type, *htaAQ*, and *clr4Δ* cells. There is no foci formation in the *htaAQ* mutant. Graph shows quantitated foci in the indicated strains. (B) Brc1 binds at γH2A sites, and Brc1 association with the telomeres and centromeres is reduced in *clr4Δ* mutants. ChIP to sites of Brc1-GFP overexpressed from the *nmt* promoter at γH2A-sites in the indicated strains. Cell cultures were asynchronous. Primer locations were described in Figure 2C and Figure 5B.

We recently discovered that γH2A is required for formation of both spontaneous and genotoxin-induced nuclear foci of Brc1, a genome maintenance protein that has a role in replication fork stability and chromatin organization [21], [23]. Spontaneous Brc1-GFP foci form in approximately 25% of wild type cells and about 60% of these foci are perinucleolar, indicating colocalization with rDNA [21]. Consistent with these observations, we detected γH2A at the rDNA in our Chip-chip analysis. Since γH2A levels were reduced in the absence of heterochromatin, we tested how deletion of Clr4 affected levels of spontaneous Brc1-GFP foci. We detected a reduction in Brc1-GFP foci in *clr4Δ* cells, from 30% to 12% (Figure 8A). To test if Brc1 binds at γH2A sites identified in this study, we performed Brc1-GFP ChIP in wild type, *htaAQ*, and *clr4Δ* mutant cells (Figure 8B). In addition to the rDNA, γH2A-dependent Brc1 binding was detected at the subtelomeres and in the outer centromere *dh* repeats (Figure 8B). In *clr4Δ* cells Brc1 binding was reduced at the centromeres and telomeres, but not at the rDNA (Figure 8B). These data show that activation of γH2A during replication recruits Brc1 to specific genomic regions, and that binding of Brc1 at centromeres and subtelomeres is associated with heterochromatin.

### Brc1 binding to γH2A is critical in the absence of Rqh1 helicase

**Figure 9:**
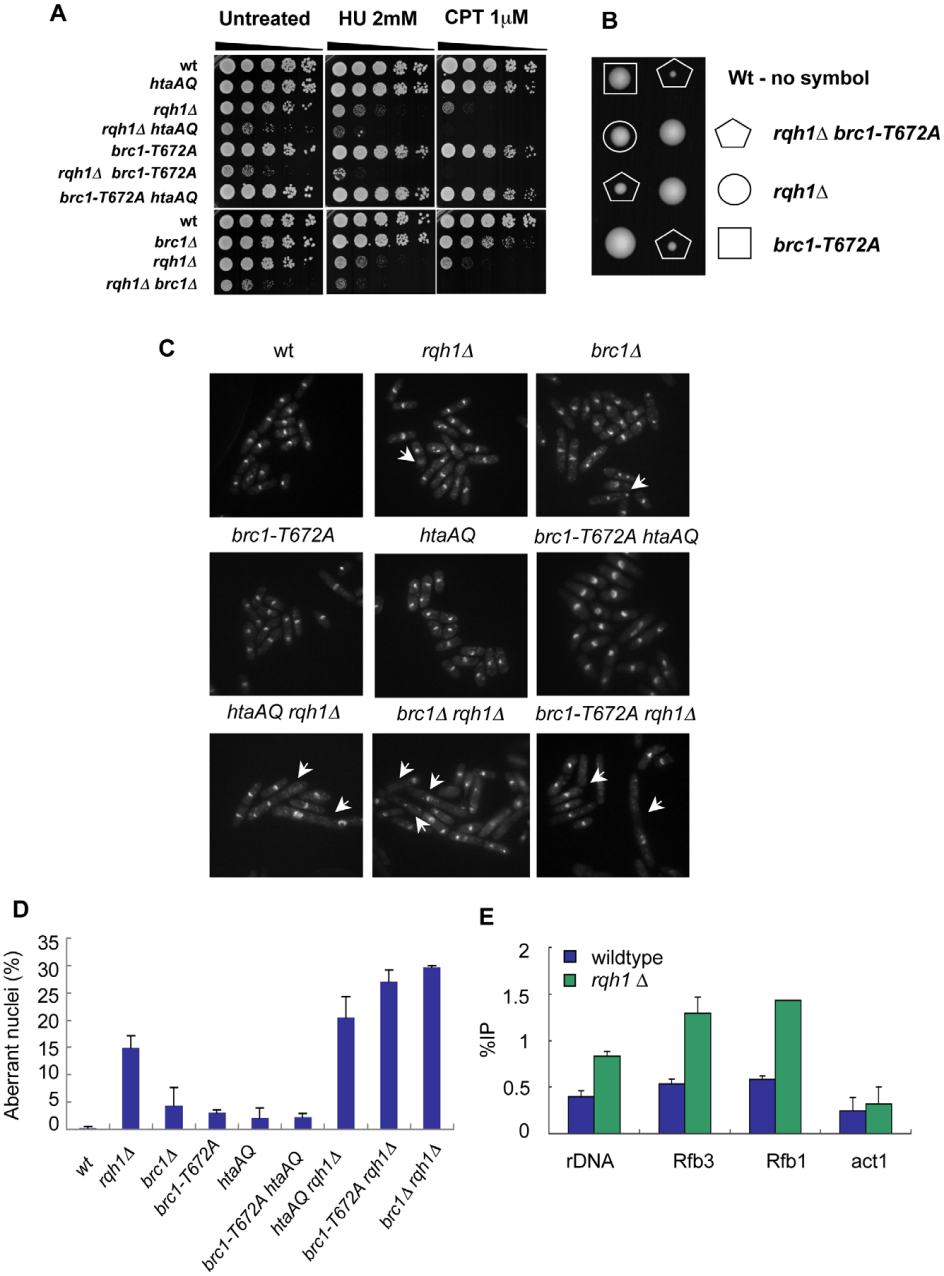
γH2A-Brc1 interactions are crucial for genome stability during unperturbed growth in the absence of Rqh1. (A) Genetic interactions of *rqh1Δ* mutant with *htaAQ, brc1Δ*, and *brc1-T672A*. Five-fold serial dilutions of indicated strains were spotted on YES medium or YES with 2 mM HU and 1 µM CPT. Pictures were taken after 3 days at 30°C. (B) Tetrad dissection of *brc1-T672A* mutant crossed with *rqh1Δ* showing two dissected asci. The double mutant (pentagon) has strong synthetic growth defects compared to either parental strain. (C) The loss of Rqh1 function in the *htaAQ*, *brc1Δ*, or *brc1-T672A* mutants leads to cell elongation, chromosome segregations defects and higher levels of aberrant mitosis (indicates by arrows) compared to the individual parental strains. The indicated strains were grown in YES media at 30°C, cells were fixed in cold 70% ethanol, stained with DAPI, and analyzed by fluorescence microscopy. (D) Quantification of (C), error bars represent the range between independent duplicate experiments. (E) γH2A levels increase at the rDNA in the absence of Rqh1. γH2A ChIP was performed in the indicated strains. Primer locations are shown in Figure 5B.

Our discovery that γH2A recruits Brc1 to critical genomic features during S-phase suggests that Rad3 and γH2A protect genome integrity in response to endogenous replication-associated DNA damage. Indeed, a function for γH2A during unperturbed growth is indicated by the increased incidence of Rad22^Rad52^ HR repair foci in *htaAQ* cells [8]. A similar increase is seen in *brc1Δ* cells [21]. However, *htaAQ* cells do not display overt growth defects that would be suggestive of severe genomic instability. It is therefore likely that γH2A works redundantly with other genome maintenance factors to protect genome integrity during S-phase. One of these factors could be Rqh1, a RecQ helicase that is required for rDNA locus stability [27], [30]. As γH2A prominently decorates the rDNA loci during S-phase, we explored the genetic interactions between *rqh1Δ* and *htaAQ* mutations. As shown in dilution assays (Figure 9A), we found that the growth of the *rqh1Δ htaAQ* cells is substantially compromised relative to either *rqh1Δ* or *htaAQ* cells [8]. These synergistic genetic interactions were maintained but apparently not enhanced in media containing hydroxyurea (HU) or camptothecin (CPT), which stall or collapse replication forks. (Figure 9A).

We next attempted to define the function of γH2A that becomes critical in the absence of Rqh1. Consistent with earlier studies [23], we found that *rqh1Δ* and *brc1Δ* had strong negative genetic interactions, similar to those between *rqh1Δ* and *htaAQ* (Figure 9A). Taken together, these data suggested that Brc1 binding to γH2A might be critical in the absence of Rqh1 activity. To address this possibility, we employed the *brc1-T672A* mutation of the BRCT_5_ domain of Brc1, which through structural, biochemical and genetic studies was shown abolish Brc1 binding to γH2A and partially compromise Brc1 function [21]. Tetrad analysis revealed strong negative genetic interactions between *rqh1Δ* and *brc1-T672A* (Figure 9B) that were confirmed in dilutions assays (Figure 9A). Microscopic examination of *rqh1Δ htaAQ, rqh1Δ brc1-T672A*, and *rqh1Δ brc1Δ* mutant cells revealed increased aberrant mitoses and chromosome segregation defects compared to parental strains (Figure 9C), which suggests that Rqh1 is required for maintaining chromosome in the *htaAQ* and *brc1-T672A* mutant backgrounds. We also confirmed by ChIP analysis that γH2A levels are elevated at the rDNA in *rqh1Δ* mutants (Figure 9E). From these data we conclude that the Rad3-γH2A-Brc1 pathway and Rqh1 DNA helicase work independently to maintain genome integrity.

## Discussion

In this study we generated a high-resolution map of γH2A during DNA replication in fission yeast. Our analysis uncovered a diverse array of γH2A-enriched regions, which included natural replication fork barriers in the mating-type locus and the tandem rDNA repeats, heterochromatin in the centromeres and telomeres, repressed subtelomeric genes and retrotransposable elements, and some repeat-rich genes. H2A phosphorylation was primarily mediated by the Rad3 checkpoint kinase, a homolog of mammalian ATR, which is required for stabilization of replication forks [3]. In both yeast and mammals, ATR^Rad3/Mec1^ is crucial for replication through genomic fragile sites, which are especially prone to replication stress [78], [79]. As the γH2A sites identified in this study all share characteristics associated with fragile sites, these results represent the first global map of replication stress regions in fission yeast. We showed that Rad3-γH2A activation at natural replication fork barriers required the Swi1-Swi3 fork protection complex, whilst γH2A formation in heterochromatin was dependent on H3K9me by the Clr4 methyltransferase. We also demonstrated that activation of γH2A during replication recruits Brc1, a factor involved in repair of stalled or collapsed replication forks [21], [23]. Our data indicates that the Rad3-γH2A-Brc1 pathway has crucial functions in maintaining genome integrity, especially in the absence of the Rqh1 helicase.

### γH2A at natural replication fork barriers

We demonstrated that γH2A is formed at natural fork barriers such as RTS1 in the mating type locus and RFBs in the rDNA. We also showed that γH2A is linked to the regulation of barrier activity by the Swi1-Swi3 fork protection complex (FPC). Deletion of Swi1 abolished γH2A formation at RTS1 in the MT locus, which indicates that the FPC triggers γH2A. Analysis of γH2A formation in the rDNA was more complicated than in the MT locus, since only three of the four rDNA barriers are dependent on the fork protection complex (RFB1, RFB2 and RFB3) but not RFP4 [51], [54]. The first three are also dependent on specific DNA-binding proteins that constitute a barrier to the advancing replication fork, Reb1 at RFB2 and RFB3, and Sap1 at RFB1 [50], [54]. However, there is no known RFP4-binding protein and this barrier is likely caused by the collision of transcription and replication machineries at the highly transcribed rDNA locus [51]. Deletion of Swi1 increases fork stalling at RFP4 and leads to an increase in Rad22 foci, which suggests elevated levels of recombination [48], [51]. Therefore, the high levels of γH2A observed at the rDNA in the absence of Swi1 may be associated with elevated recombination, suggesting that γH2A is a marker of recombination. This could also explain why we detected γH2A near the tDNA barriers only when Swi1 was deleted. Similar to RFP4, the tDNAs are nonpolar fork barriers triggered by collisions between the replisome and transcription machinery [56]. The FPC suppresses recombination at the tDNAs and facilitates replisome progression through these sites [56]. The increase in γH2A at the tDNAs in *swi1Δ* cells again suggests that γH2A is associated with “hot-spots” of recombination. This observation is supported by our studies of γH2A formation at an ectopic RTS1 replication fork barrier inserted between two direct repeats of *ade6* alleles [49]. We observed high levels of γH2A on the side where fork stalling occurs and recombination is initiated, which occurs in the absence of DNA DSBs[49]. Additionally, we observed an asymmetric distribution of γH2A at RTS1, which contrasts the typical bimodal γH2A distribution found around DSBs [10]. Altogether, our data show that γH2A is triggered at natural replication fork barriers and suggests that phosphorylation of H2A is associated with recombination hotspots at stalled replication forks.

### Heterochromatin-dependent γH2A formation

The enrichment of γH2A in heterochromatin regions during DNA replication is the most intriguing outcome of our ChIP-on-chip analysis. We demonstrated that γH2A precisely colocalizes with Clr4-dependent H3K9me in the pericentromeric *dg/dh* repeats, the subtelomere, and IR elements flanking the MT silent region. We also detected γH2A in transcriptionally repressed subtelomeric regions and in retrotransposons, which are silenced by Clr3/Clr6-dependent histone deacetylation. Importantly, the formation of γH2A at the centromeres and in subtelomeric regions was dependent on the Clr4 methyltransferase, which mediates the crucial steps in heterochromatin establishment [80]. Our data suggests that either the active process of heterochromatin establishment or the resulting heterochromatin structure lead to replication fork pausing, stalling or collapse, which triggers recruitment of Rad3 and γH2A formation.

Heterochromatin establishment occurs during DNA replication and involves assembly of multiple protein-DNA complexes, which could impede replisome progression [69], [81], [82]. Additionally, RNAi transcription occurs at the same time at the *dg/dh* repeats in the centromere and subtelomeres, which could induce collisions between the replisome and transcription machinery [81]–[83]. How replication fork progression is coordinated with heterochromatin formation is poorly understood. Since Swi6 facilitates early origin firing at the centromere *dg/dh* repeats [74], heterochromatin is at least partially present when replication begins. Replication fork pausing has been detected in budding yeast centromeres [84] and structures suggestive of replication intermediates have been reported in fission yeast centromeres although no specific replication fork pause sites have been identified [85].

Replication fork pausing also occurs at the telomeres and is increased in the absence of Taz1 [76]. Thus, we were surprised that γH2A levels in the subtelomeres of *taz1Δ* mutants remained similar to wild type cells. We showed that γH2A forms in subtelomeric heterochromatin, and is rapidly depleted below background levels in the TAS region and telomere repeats, where Taz1 binds. This suggests that the transition from the subtelomere into the TAS is accompanied by a change in chromatin structure, which is consistent with reports that *S. pombe* telomeres may be non-nucleosomal or contain alternative nucleosomal configurations [75], [77]. The DNA fragments containing stalled forks that were analyzed in *taz1Δ* mutants were located outside the subtelomeric γH2A domain [76]. Our data strongly suggests that γH2A formation in the subtelomeres is linked specifically to the presence of heterochromatin, independently of Taz1. This is supported by observations that deletion of Taz1 does not change existing heterochromatin levels at the subtelomeres [77] and our data that γH2A formation is associated with Clr4 activity.

There is accumulating evidence that replication of heterochromatin requires extra vigilance by genome maintenance factors [86], [87]. Deletion of Rad3 destabilizes the centromeres and telomeres, resulting in increased gross chromosomal rearrangements [42], [43], [86]. We detected formation of γH2A at the subtelomeres in G2 phase in *rad3Δ*, which indicates unrepaired DNA damage, likely a result of telomere shortening through recombination [42]. At the centromeres γH2A formation temporally and physically coincides with recruitment of the Rad51 recombinase in early S-phase, where it is required for suppression of gross chromosomal rearrangements between centromere repeats [86]. The Smc5/6 complex, an essential factor for genome stability, also binds to heterochromatin regions during S-phase, where it is thought to suppress recombination [87]. Put together, formation of γH2A in heterochromatin coincides with DNA replication, and participates in either maintenance of replication fork stability, recombination, or repair of collapsed replication forks. This role may be partially conserved in mammals during replication of specialized chromatin domains such as the silent X chromosome, where γH2AX and BRCA1 foci form in S-phase [88].

### Genetic interactions between Rad3-γH2A-Brc1 pathway and Rqh1 DNA helicase are required for maintaining genomic stability

A number of genetic studies support the idea that γH2A has a role in repair of replication-associated DNA damage, including our investigations of γH2A binding to Brc1 [21]. Brc1 binds to γH2A in response to endogenous replication stress and DNA damaging agents via its C-terminal BRCT domain. In this study we demonstrated that Brc1 is recruited by γH2A to the rDNA, centromeres, and telomeres. Since γH2A forms transiently during S phase, Brc1 recruitment suggests that these regions undergo DNA damage during replication. A majority of spontaneous γH2A-dependent Brc1 foci colocalize with the rDNA [21] and Brc1 is needed for viability in the absence of the Rqh1 helicase, which functions to maintain rDNA stability [23]. We showed that the *rqh1Δ brc1-T672A* mutant, in which Brc1 binding to γH2A is abolished, and the *htaAQ rqh1Δ* mutants have poor viability and severe chromosome segregation defects. Along with these genetic interactions, the increased γH2A levels in the rDNA of *rqh1Δ* mutants support the idea that γH2A may function in the maintenance of rDNA in a pathway mediated by Brc1. Brc1 association with heterochromatin in the centromeres and telomeres indicates that the Rad3-γH2A-Brc1 pathway also functions to prevent DNA damage in heterochromatin. Interestingly, deletion of Brc1 is synthetic lethal with mutants of the Smc5/6 complex, which suppresses recombination in rDNA and heterochromatin [22], [89]–[91]. It is noteworthy that the binding of Smc5/6 to centromeric heterochromatin is decreased in Clr4Δ cells [87], similar to our observations of Brc1. Our ideas are supported by studies of the putative Brc1 homolog in *S. cerevisiae*, Rtt107/Esc4, which is involved in DNA repair at stalled replication forks, maintenance of rDNA stability, and chromatin silencing [23], [92]–[96]. PTIP, a mammalian protein that is structurally related to Brc1, has roles in both transcriptional regulation and maintenance of genomic stability [97]. The role of PTIP in the response to DNA damage is poorly understood, although there are indications that it participates in homologous recombination [98]. The recruitment of Brc1 to γH2A-sites during replication suggests that it and possibly Rtt107 or PTIP may function in maintaining genomic integrity in regions that are susceptible to replication stress, particularly the rDNA and heterochromatic loci. The recruitment of Brc1 to these sites is crucial in the absence of the Rqh1 RecQ helicase. It will be interesting to determine if γH2AX similarly recruits PTIP to fragile genomic sites in mammalian cells.

### Comparison of γH2A landscape between the fission and budding yeasts

The genome-wide distribution of γH2A in budding yeast was recently described [31] and allows us to compare genome protection mechanisms between the two yeast species. The patterns of γH2A formation are remarkably similar between these highly divergent organisms. Both studies identified γH2A domains at natural replication fork barriers, centromeres and telomeres, and repressed chromatin. This indicates that the functions of γH2A in genome stability in these regions are likely to be conserved in higher eukaryotes.

Several γH2A sites found in budding yeast were absent in fission yeast, including tDNAs, LTRs, and replication origins. Fission yeast tDNAs are weak, nonpolar fork barriers [56], and our data indicates that the Swi1-Swi3 complex mediates replication through these barriers in a manner that avoid triggering Rad3 activation, as evidenced by lack of γH2A at these sites. We identified γH2A near tDNAs only at the centromeres, where these elements are boundaries to heterochromatin spreading and regulate centromere functional organization in fission yeast [45], [58]. Similarly, we observed association of γH2A with LTRs only where they flank Tf2 retrotransposons. These LTRs recruit CenpB-family proteins to assemble repressive structures called ‘Tf-bodies’[99], which may impede replication fork progression and lead to γH2A formation. We did not observe γH2A loading at replication origins, where peaks of γH2A were detected in budding yeast. Fission yeast origins are more similar to mammalian cells as they are inefficient and lack strong regulation, whereas in budding yeast there are specific origins programmed to fire efficiently in S phase [100]. Due to the stochastic origin activity in fission yeast, the likelihood of fork stalling at a specific origin is low, and the accumulation of γH2A near origins may not be detectable in an average cell population.

The most fascinating discovery of both studies is that γH2A associates with heterochromatic regions and repressed genes. This is interesting considering that the pathways of heterochromatin establishment in these yeasts are highly divergent [101]. In budding yeast gene silencing occurs primarily through histone deacetylation, but in *S. pombe* there are additional pathways that involve Clr4-dependent histone methylation, recruitment of HP-1 homologs, and RNAi transcription [101], [102]. Heterochromatin structure of *S. pombe* is more similar to mammalian heterochromatin, particularly at the centromeres [101]. In *S. pombe* subtelomeres and centromeres γH2A forms transiently during DNA replication, but in *S. cerevisiae* γH2A is constitutively present in the subtelomeres, and transiently forms in the centromeres. Although there are indications that γH2AX has a role in chromatin silencing and chromosome segregation in other organisms [103], [104], neither yeast study was able to link γH2A to a heterochromatin-specific function. Put together, these data strongly suggest that repressed chromatin impedes replication fork progression, and that Rad3-γH2A activation may have a critical role in replication of heterochromatin regions in higher eukaryotes.

### Conclusion

During DNA replication the genome is very vulnerable to mutations and gross chromosomal rearrangements [1]. We demonstrated that genome-wide mapping of γH2A is a sensitive method for identifying regions of DNA replication stress. We also showed that γH2A may play a role in stabilizing replication forks stalled by natural impediments, such as RFBs, and that γH2A is linked to heterochromatin establishment during replication. γH2A-dependent Brc1 binding is a link between γH2A formation and mechanisms that ensure genome stability during DNA replication. Since γH2A modification in heterochromatin regions is conserved among several organisms, it is plausible that this modification has a yet-undiscovered role in heterochromatin function, which is difficult to detect due to redundant genetic pathways that compensate in its absence. ChIP-on-chip analysis of γH2A in a genetically tractable organism such as fission yeast will be a useful assay for studying genome-wide effects of mutations that impair DNA replication or checkpoint pathways.

## Materials and Methods

### Yeast strains and methods

Fission yeast strain genotypes are listed in Table S2. Standard fission yeast methods were used as described previously [39]. Gene deletions and epitope-tagging were performed as described [105]. For synchronization of cells using *cdc25-22* block and release, cells containing the temperature sensitive *cdc25-22* allele were incubated at restrictive temperature (36°C) for 4 hours to arrest the cell cycle in G2 [39]. Upon release to permissive temperature (25°C), the cells synchronously enter the cell cycle. Progression into S-phase was monitored microscopically by counting cells that contained septa, the appearance of which correlates with S-phase [39]. Cells were fixed for ChIP experiments when the septation index was between 60–80%. Ectopic expression of *pRep41-N-GFP-brc1^+^* for microscopy and ChIP analysis was under the control of the thiamine-repressible *nmt41* promoter. Induction of plasmid expression was performed in selective medium in the absence of thiamine for 18–20 hours.

### Western blot analysis

For γH2A and H2A Western blotting, histone-enriched protein extracts were prepared as previously described in [33]. Polyclonal anti-γH2A antibody (courtesy of C. Redon) was used for γH2A detection. Polyclonal anti-H2A antibody (07–146, Millipore) was used for H2A detection. Images were acquired on the Odyssey Infrared Imaging System (LI-COR Biosciences).

### Microscopy

Cells were grown in YES liquid medium at 30°C, washed once in 1x PBS and imaged live for Brc1-GFP foci, or fixed overnight in cold 70% ethanol, washed once in 1x PBS, and mixed with 500 ug/ml DAPI for analysis of chromosome segregation defects. Cells were photographed using a Nikon Eclipse E800 microscope equipped with a Photometrix Quantix charge-coupled device camera. Error bars represent the standard deviation between 3 independent experiments, or range for 2 independent experiments.

### ChIP assay

ChIP experiments were performed as described [20] with the following modifications. Cleared lysates were incubated with protein G Dynabeads (Invitrogen) pre-bound to anti-γH2A antibody (Courtesy of C.Redon) or anti-GFP antibody (Roche). DNA was purified using the QIAquick PCR Purification Kit (Quiagen). Quantitative PCR was performed on input and ChIP samples using the Chromo4 Real-Time PCR Detection System (Bio-Rad) using the iQ SYBR Green Supermix (Bio-Rad). QPCR primers are listed in Table S3 with the exception of the subtelomere primers, which are from [70] and tDNA primers, which are from [87]. Percent of immunoprecipitated DNA (%IP) in the ChIP samples was calculated relative to the amount of DNA in the input samples. ChIP fold enrichment was calculated relative to actin. All error bars represent the standard error between experimental triplicates.

### ChIP-on-chip analysis

Input and ChIP DNA samples were prepared using standard Affymetrix protocols and hybridized on Affymetrix *S. pombe* tiling arrays FR1.0. See Supporting Information (Text S1) for description of MAT score and other methods.

## Supporting Information

Figure S1 γH2A forms at HO-induced DNA double-strand breaks. ChIP-qPCR analysis of γH2A distribution at an HO endonuclease-induced DNA double-strand break was performed in the indicated strains. As a control ChIP was performed in conditions when HO endonuclease expression was suppressed (No DSB) and in an *htaAQ* mutant that cannot be phosphorylated. ChIP enrichment was quantitated as percent immunoprecipitated DNA compared to input (%IP). The primer locations are indicated as distance in kilobases (kb) from the HO-induced DSB site.
(0.04 MB PDF)

Figure S2 γH2A formation is abolished in the absence of both Rad3 and Tel1. ChIP-qPCR analysis was performed in asynchronous cultures of the indicated strains. ChIP enrichment was quantitated as %IP (y-axis).
(0.04 MB PDF)

Figure S3 Example of γH2A ChIP-on-chip distribution in retrotransposons, wtf elements, and a gene with repetitive DNA sequences. (A) Detailed distribution of γH2A in Tf2 retrotransposons (Tf2–5 and Tf2–6). Enrichment of γH2A on the graph is shown as MAT score (y-axis). Chromosome annotation and coordinates (x-axis, in kilobases, (kb)) downloaded from the *S.pombe* Genome Project (Sanger Center: www.sanger.ac.uk/Projects/S_pombe/). Vertical black rectangles (below graph) represent gene locations. (B) Detailed distribution of γH2A in wtf repeats (wtf19, wtf20). (C) Distribution of γH2A in gene SPBPJ4664.02.
(0.07 MB PDF)

Figure S4 Absence of γH2A does not affect cohesin levels at the centromeres and telomeres. ChIP-qPCR analysis of Rad21-3HA was performed in wildtype and *htaAQ* mutants, and an untagged strain was used as a negative control. Cells were synchronized using *cdc25-22* block and release and ChIP samples were collected in S-phase, as determined by septation index.
(0.03 MB PDF)

Figure S5 Taz1 distribution in the telomeres. ChIP-qPCR analysis of Taz1-3HA was performed in asynchronous wildtype cultures and an untagged strain was used as a negative control. Primers are described in the text.
(0.03 MB PDF)

Table S1 List of all genes and features containing phospho-H2A in the ChIP-on-Chip analysis.
(0.05 MB XLS)

Table S2 *S. pombe* strains used in this study.
(0.03 MB DOC)

Table S3 QPCR primers used in this study.
(0.03 MB DOC)

Text S1 Supporting methods.
(0.05 MB DOC)
