# Supplementary figures and images for: Rad3ATR Decorates Critical Chromosomal Domains with γH2A to Protect Genome Integrity during S-Phase in Fission Yeast
Source: PLoS Genet. 2010 Jul 22;6(7):e1001032. doi: 10.1371/journal.pgen.1001032 (PMC2908685; doi:10.1371/journal.pgen.1001032)

S1

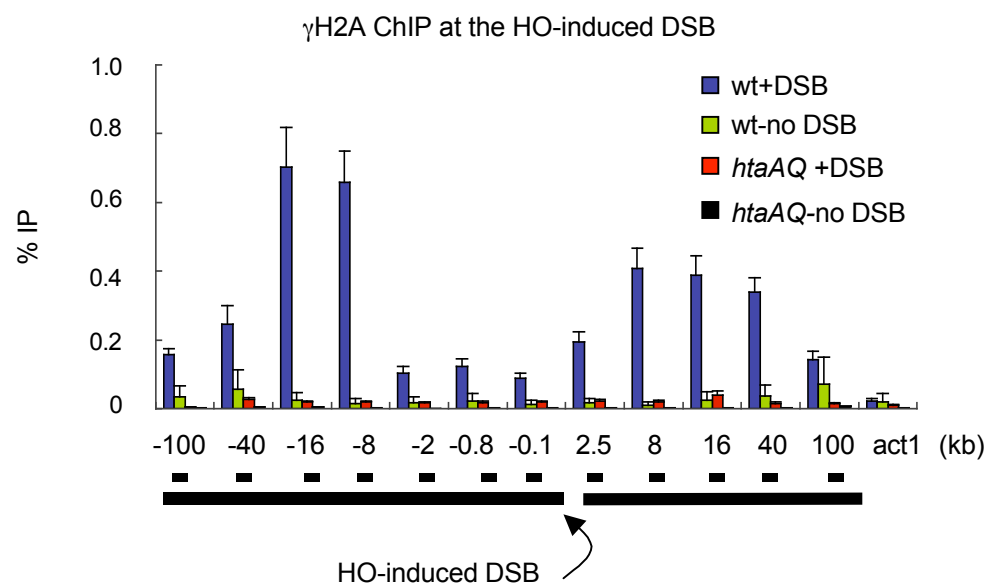

Supplement: Figure S1 — γH2A forms at HO-induced DNA double-strand breaks. ChIP-qPCR analysis of γH2A distribution at an HO endonuclease-induced DNA double-strand break was performed in the indicated strains. As a control ChIP was performed in conditions when HO endonuclease expression was suppressed (No DSB) and in an htaAQ mutant that cannot be phosphorylated. ChIP enrichment was quantitated as percent immunoprecipitated DNA compared to input (%IP). The primer locations are indicated as distance in kilobases (kb) from the HO-induced DSB site. (0.04 MB PDF) [file pgen.1001032.s001.pdf]

S2

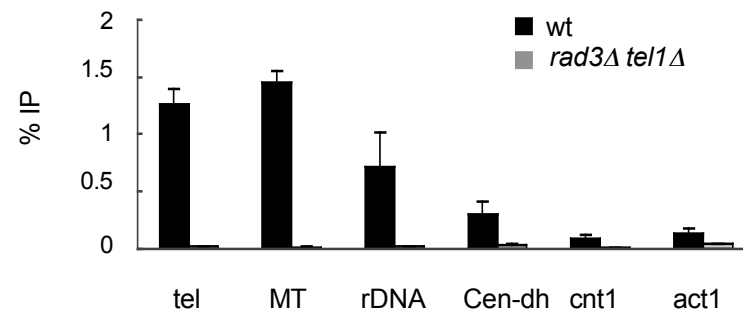

Supplement: Figure S2 — γH2A formation is abolished in the absence of both Rad3 and Tel1. ChIP-qPCR analysis was performed in asynchronous cultures of the indicated strains. ChIP enrichment was quantitated as %IP (y-axis). (0.04 MB PDF) [file pgen.1001032.s002.pdf]

S4

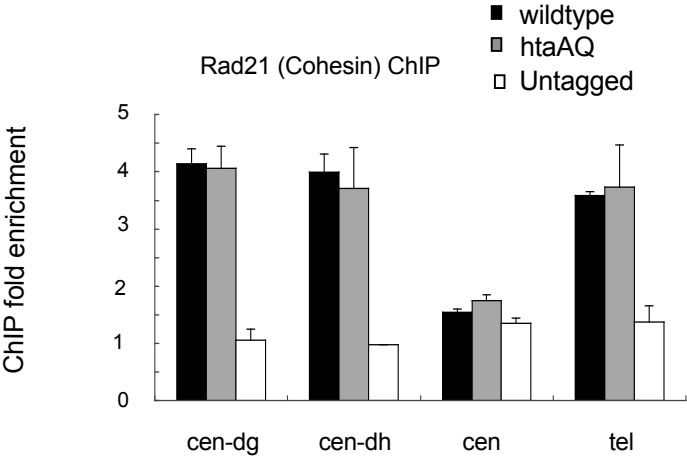

Supplement: Figure S4 — Absence of γH2A does not affect cohesin levels at the centromeres and telomeres. ChIP-qPCR analysis of Rad21-3HA was performed in wildtype and htaAQ mutants, and an untagged strain was used as a negative control. Cells were synchronized using cdc25-22 block and release and ChIP samples were collected in S-phase, as determined by septation index. (0.03 MB PDF) [file pgen.1001032.s004.pdf]

S5

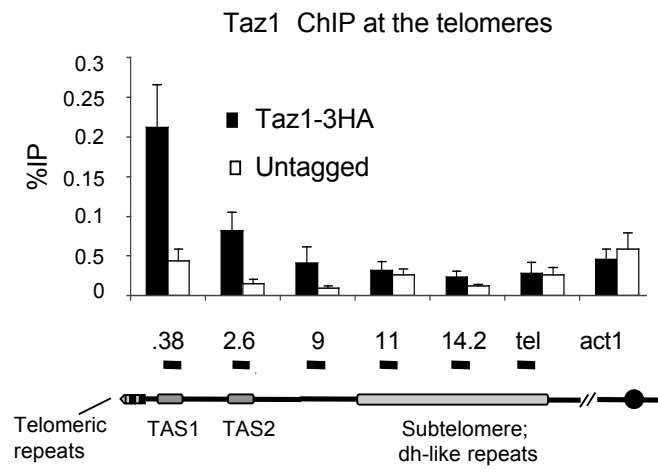

Supplement: Figure S5 — Taz1 distribution in the telomeres. ChIP-qPCR analysis of Taz1-3HA was performed in asynchronous wildtype cultures and an untagged strain was used as a negative control. Primers are described in the text. (0.03 MB PDF) [file pgen.1001032.s005.pdf]
