# Supplementary material for: Rad3ATR Decorates Critical Chromosomal Domains with γH2A to Protect Genome Integrity during S-Phase in Fission Yeast
Source: PLoS Genet. 2010 Jul 22;6(7):e1001032. doi: 10.1371/journal.pgen.1001032 (PMC2908685; doi:10.1371/journal.pgen.1001032)

S3

Example of γH2A distribution in Tf2 retrotransposons

A

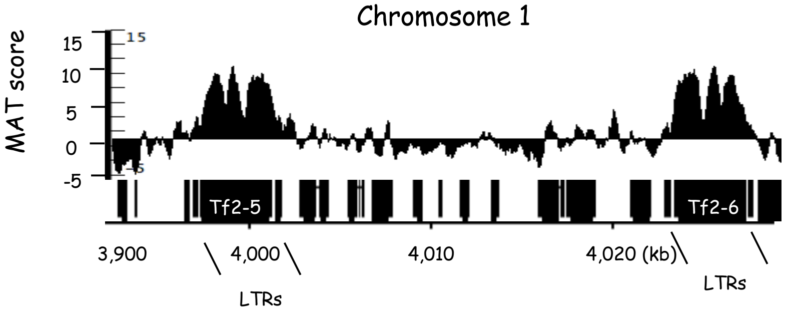

B

Example of γH2A distribution in *wtf* elements

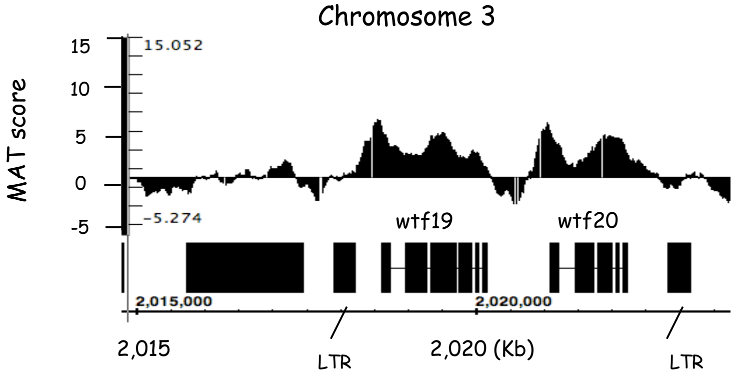

C

γH2A distribution in a repetitive gene, SPBPJ4664.02

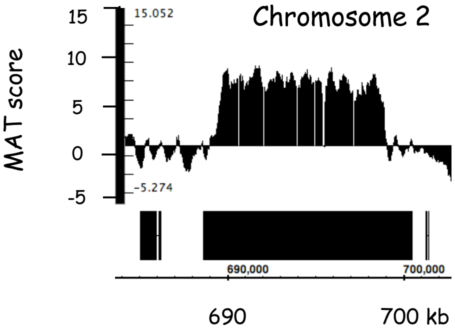

Supplement: Figure S3 — Example of γH2A ChIP-on-chip distribution in retrotransposons, wtf elements, and a gene with repetitive DNA sequences. (A) Detailed distribution of γH2A in Tf2 retrotransposons (Tf2–5 and Tf2–6). Enrichment of γH2A on the graph is shown as MAT score (y-axis). Chromosome annotation and coordinates (x-axis, in kilobases, (kb)) downloaded from the S.pombe Genome Project (Sanger Center: www.sanger.ac.uk/Projects/S_pombe/). Vertical black rectangles (below graph) represent gene locations. (B) Detailed distribution of γH2A in wtf repeats (wtf19, wtf20). (C) Distribution of γH2A in gene SPBPJ4664.02. (0.07 MB PDF) [file pgen.1001032.s003.pdf]
