# Supplementary material for: Rad3ATR Decorates Critical Chromosomal Domains with γH2A to Protect Genome Integrity during S-Phase in Fission Yeast
Source: PLoS Genet. 2010 Jul 22;6(7):e1001032. doi: 10.1371/journal.pgen.1001032 (PMC2908685; doi:10.1371/journal.pgen.1001032)
Supplement: Table S2 — S. pombe strains used in this study. (0.03 MB DOC) [file pgen.1001032.s007.doc]

**Table S2. S.pombe strains used in this study**

| Strain# | Genotype | Source |
| --- | --- | --- |
| LLD3716 | *h- ura4-D18 his3-D1 arg3::HO site-kanMX6 rad22-2xCFP-kanMX6 ars1::nmt41promoter-HO-his3+ crb2-D2::ura4+ leu1-32::TAP-crb2-leu1+* | Lab strain |
| LLD3717 | *h- ura4-D18 his3-D1 arg3::HO site-kanMX6 rad22-2xCFP-kanM6X ars1::nmt41promoter-HO-his3+ crb2-D2::ura4+ leu1-32::TAP-crb2-leu1+ hta1-S129A::ura4+ hta2-S128A::kanMX6* | Lab strain |
| MCW1262 | *h- ura4-D18 leu1-32 his3-D1 arg3-D4 ade6-M375 int::pUC8/his3+/RTS1 site A orientation 1/ade6-L469* | M.Whitby |
| MCW1433 | *h- ura4-D18 leu1-32 his3-D1 arg3-D4 ade6-M375 int::pUC8/his3+/RTS1 site A orientation 2/ade6-L469* | M.Whitby |
| OL305 | *h- leu1-32 ura4-D18 rad3::kanMX tel1::hphMX6* | O.Limbo |
| OL1157 | *h+ leu1-32 ura4-D18 rqh1::ura4+* | O.Limbo |
| PR37 | *h-* | Lab strain |
| PR109 | *h- leu1-32 ura4-D18* | Lab strain |
| PR2700 | *h? leu1-32 ura4-D18 ade6-? his-? cds1::ura4 cdc25-22* | Lab strain |
| PR3650 | *h- leu1-32::2xYFP-crb2-leu1+ ura4-D18 his3-D1 arg3::HOsite-kanMX4 ars1(MluI)::nmt41-HO-his3+ crb2-D2::ura4 rad22-2xCFP-kanMX6* | Lab strain |
| PR3652 | *h- leu1-32::2xYFP-crb2-leu1+ ura4-D18 his3-D1 arg3::HOsite-kanMX4 ars1(MluI)::nmt41-HO-his3+ crb2-D2::ura4 rad22-2xCFP-kanMX6 hta1-S129A::ura4+ hta2-S128A::his3+* | Lab Strain |
| PR3302 | *h- leu1-32 ura4-D18 ade6-M210 his3-D1 rqh1::ura4+ hta1-S129A::ura4+ hta2-S128A::his3+* | Lab strain |
| PR4363 | *h- leu1-32 ura4-D18 his3-D1 cdc25-22* | Lab strain |
| PR4408 | *h90 (h+) mat1-P mat2,3::leu2 leu1-32 cdc25-22* | Lab strain |
| PR4410 | *h90(h-) mat1-M smt0 mat2,3::Leu2 leu1-32 cdc25-22* | Lab strain |
| PR4673 | *h+ leu1-32 brc1-wt-2GFP:hphMX6* | Lab strain |
| PR4675 | *h+ leu1-32 brc1-T672A-2GFP:hphMX6* | Lab strain |
| PR4730 | *h+ leu1-32 ura4-D18 brc1::hphMX6 rqh1::kanMX6* | Lab strain |
| PR4744 | *h- leu1-32 ura4-D18 his3-D1 brc1-T672A-2GFP:HphMx hta1-S129A::ura4+ hta2-S128A::his3+* | Lab strain |
| SR139 | *h+* *leu1-32 ura4-D18 hta1-S129A::ura4+ hta2-S128A:kanMX6* | This study |
| SR155 | *h- leu1-32 ura4-D18 hta1-S129A::ura4+ hta2-S128A:kanMX6* | This study |
| SR172 | *h- leu1-32 ura4-D18 ade6-M210 his3-D1 tel1::kanMX4 cdc25-22* | This study |
| SR185 | *h- leu1-32 ura4-D18 rad3::kanMX4 cdc25-22* | This study |
| SR222 | *h- leu1-32 ura4-D18 swi1::kanMX6 cdc25-22* | This study |
| SR225 | *h- leu1-32 ura4-D18 swi3::kanMX6 cdc25-22* | This study |
| SR275 | *h- leu1-32-ura4-D18 clr4::natMX6* | This study |
| SR285 | *h- ura4-D18 leu1-32 ade6-M210 his3-D1 clr4::kanMX6 cdc25-22* | This study |
| SR242 | *h+ leu-32 ura4-D18 brc1::natMX6* | This study |
| SR413 | *h+ leu-32 ura4-D18 rqh1::ura4+ brc1-T672A-2GFP:hphMX6* | This study |
| SR441 | *h+ leu1-32 ura4-D18 pRep41-N-GFP-brc1^+^* | This study |
| SR442 | *h+ leu1-32 ura4-D18 clr4::natMX6 pRep41-N-GFP-brc1^+^* | This study |
| SR443 | *h- leu1-32 ura4-D18 hta1-S129A::ura4+ hta2-S128A:kanMX6 pRep41-N-GFP-brc1^+^* | This study |
