# Supplementary material for: Rad3ATR Decorates Critical Chromosomal Domains with γH2A to Protect Genome Integrity during S-Phase in Fission Yeast
Source: PLoS Genet. 2010 Jul 22;6(7):e1001032. doi: 10.1371/journal.pgen.1001032 (PMC2908685; doi:10.1371/journal.pgen.1001032)
Supplement: Table S3 — QPCR primers used in this study. (0.03 MB DOC) [file pgen.1001032.s008.doc]

**Table S3. Primer Sequences.**

| act1 | F, 5’-CGC CGA ACG TGA AAT TGT TCG TGA-3’  R, 5’- AAG GGA GGA AGA TTG AGC AGC AGT -3’ |
| --- | --- |
| cen | F, 5'- CAA CCG TTG CAA CTT ACA TCA GCA-3'  R, 5'- CCG GTC GCC AAA TAG CAA TGA GAT-3' |
| cen-dh | F, 5'- TGC TGT CAG CTC ACT CAA GTC CAA-3'  R, 5'- AGG ACT AAG CCC AAG CAC CGT ATT-3' |
| cen-dg | F, 5'- TAC CGT GAT TAG CCT TAC TCC GCA-3'  R, 5'- ACC GCA AGA TAG AGT AGG ATG GGT-3' |
| imr | F, 5' - AGT GGA CAA GCC AGA ACT CGA ATC - 3'  R, 5’ - TCA ACC ATG GGC ATG TGG TGT AGA - 3' |
| rDNA | F, 5’- GGA CGG TGG CCA TGG AA-3’  R, 5’-CAT TCG GCC GGT GAG TTG-3’ |
| Rfb3 | F, 5’-ACG CCG GTG AAA TAC CAC TAC CTT-3’  R, 5’-GAG GAT CAG TTC GCG AAG AAA CTT-3’ |
| Rfb2 | F, 5’-AGT GCA AGG AGC TAT CTT GGT GGT-3’  R, 5’-CGT GTG TGT GTG TGT GTG TGT TGT-3’ |
| Rfb1-1 | F, 5’-CAT GTA GCT GAA GCC AGA GTG CAA-3’  R, 5’-TCT CTC CAC CTT CCC ATA ACA TGC-3’ |
| Rfb1-2 | F, 5’-AGA AGT AAG GAG GGA GTA GTG GGA-3’  R, 5’-CGT CCA TTT CGG TGC CTA TTT CCT-3’ |
| tel | F, 5’- TCA AAG TTG GCG ACG TTG CTG ATG-3’  R, 5’- AAG CAA TGT GTG GAG CAA CAG TGG-3’ |
|  | MT locus primers |
| -8kb | F, 5’- AGG CCT AGG TAA AGG TTT GTG GGT-3’  R, 5’-CGC AAG GAC AAT TGC CCA TCC AAA-3’ |
| -3kb | F, 5’- GCA GCA CGG TTT GAG TTG ATG CTA-3’  R, 5’- TCC CAT CCA GAC GAC AAC GAT TCA-3’ |
| 1.5kb | F, 5’- TCA AAC CTT CCT CCT ACA CGC CAA-3’  R, 5’- AAA GGT TTG CAC GGG ATG ATA GCG-3’ |
| 5kb /MT | F, 5’- AGC ACG CAC CCT ACG AAG GAA TTA-3’  R, 5’- GCA GCG TCT GAA AGC TTC TGT GAT-3’ |
| 14kb | F, 5’- CCA TCA GCT CAT CTG CGA ACT TGT-3’  R, 5’- TCA TTG AGC AGC GGT TCG ATT TGC-3’ |
| 17kb | F, 5’- AAG GAG ACT GTG TTT CAC CTC CGT-3’  R, 5’- GCC CGC CTT CTC AAA CAA TCT CAA-3’ |
|  | Ectopic RTS1 primers |
| -3kb | F, 5’-ATA CGG GAT AAT ACC GCG CCA CAT-3’  R,5’-TGC ACG AGT GGG TTA CAT CGA ACT-3’ |
| -1.5kb | F,5’-GTG CCA ACA GTG ATA CGC AAT GCT-3’  R,5’-AAA TGG CCA CGA TGC ACA AAG TCG-3’ |
| -1kb | F,5’-ACA TTC CGT ACG AAG GTG GAC ACA-3’  R,5’-CAT GGG TGT TGG CAG CGA TGA AAT-3’ |
| 0.3kb | F,5’-TGA TGG AGG ACG TGA GCA CAT TGA-3’  R,5’-TTG AAT GCA TCG CAG AGT TGC AGG-3’ |
| 1kb | F,5’-ACA CCC AAC TCT CGA CTT CCA CAA-3’  R,5’-AGA TGC AAA GTT GCA CCG GGA ATG-3’ |
| 1.5kb | F,5’-TAT GCT CGT ACC GCA GCT TCA AGA -3’  R,5’- GCA GCA ACC ATA CCA GGC AAA TGA-3’ |
| 3kb | F,5’-TGC TGT TGA GAG CTG CAA CGA ATG-3’  R,5’-ACA CCA ATT CAT GCC GAT GGT GAC-3’ |
| 5kb | F,5’-TCC GTG CGC CTT ACA CTT CTA ACA-3’  R,5’-GCG CTG ATT TGG TTG CTT GAA TGC-3’ |
