# Supplementary material for: Rad3ATR Decorates Critical Chromosomal Domains with γH2A to Protect Genome Integrity during S-Phase in Fission Yeast
Source: PLoS Genet. 2010 Jul 22;6(7):e1001032. doi: 10.1371/journal.pgen.1001032 (PMC2908685; doi:10.1371/journal.pgen.1001032)
Supplement: Text S1 — Supporting methods. (0.05 MB DOC) [file pgen.1001032.s009.doc]

**Supporting information**

**Supporting methods**

**ChIP-on-chip analysis:**

Chip and Input DNA samples were amplified by random-priming PCR using a standard GeneChIP ChIP-on chip protocol from Affymetrix (available at [www.affymetrix.com/support](http://www.affymetrix.com/support)). DNA was quantitated on the Nanodrop ND-1000. DNA (7.5ug) was fragmented and labeled using the GeneChIP WT Double-Stranded DNA Terminal Kit ( P/N 900812). Samples were checked by gel-shift assay to assess labeling efficiency and hybridized overnight to the S. pombe Tiling 1.0FR Array. Hybridization and scanning of samples was performed using standard Affymetrix protocols and reagents from the GeneChIP Hybridization, Wash and Stain Kit (P/N 900720). Chips were screened on the Affymetrix GeneChIP Scanner 30007G with default settings and a target intensity of 250 for scaling. Data analysis was performed using the Model-based Analysis of Tiling arrays (MAT) algorithm [1]. This algorithm was designed specifically for ChIP-on-chip data analysis using tiled microarrays. First, a baseline probe behavior model is built based on the nucleotide sequence and copy number of each probe on the array. This model is used to standardize the intensity values for each individual probe and calculate values for probe signals of the ChIP samples, which are output as MAT scores. A sliding window is used to score the data. The Input DNA was used as a control, whereby the MAT score of the Input was subtracted from the MAT score for the IP in each window. The MAT algorithm allows robust *P* value and false discovery rate calculations to calculate ChIP enrichment. *P* values were assigned by estimating the non-enriched null distribution of the MAT scores. The MAT score cutoff can be set by the user based on qPCR measurements or be determined by FDR or a *P* value. Parameters used for our data analysis were: Bandwidth = 200; MaxGap=200; MinProbe=11; p-value cutoff=1e-5. ChIP enrichment is represented as MAT score in the graphs. The MAT scores were mapped to fission yeast genomic positions using the Sanger 8/23/07 library for S. pombe.

**ChIP analysis at an HO-induced DSB:**

Strains LLD3716 and LLD3717 were used to analyze formation of γH2A at an HO endonuclease-induced DSB using methods previously developed in our laboratory [2]. Briefly, the HO site is located in the Arg3 locus and the HO-endonuclease expression is regulated by induction of a thiamine-repressible promoter. Samples were collected at 25 hours after removal of thiamine. ChIP was performed as described in the Materials and Methods section. QPCR primer sequences are available upon request.

**Supporting References:**

1. Johnson WE, Li W, Meyer CA, Gottardo R, Carroll JS, et al. (2006) Model-based analysis of tiling-arrays for ChIP-chip. PNAS 103: 12457-12462.

2. Du L-L, Nakamura TM, Russell P (2006) Histone modification-dependent and -independent pathways for recruitment of checkpoint protein Crb2 to double-strand breaks. Genes Dev 20: 1583-1596.
